# Development of Transgenic Cotton Lines Expressing *Allium sativum* Agglutinin (ASAL) for Enhanced Resistance against Major Sap-Sucking Pests

**DOI:** 10.1371/journal.pone.0072542

**Published:** 2013-09-04

**Authors:** Chakravarthy S. K. Vajhala, Vijaya Kumar Sadumpati, Hariprasad Rao Nunna, Sateesh Kumar Puligundla, Dashavantha Reddy Vudem, Venkateswara Rao Khareedu

**Affiliations:** 1 Centre for Plant Molecular Biology, Osmania University, Hyderabad**,** Andhra Pradesh, India; 2 Nuziveedu Seeds Ltd, Ranga Reddy, Andhra Pradesh, India; Wuhan Bioengineering Institute, China

## Abstract

Mannose-specific *Allium sativum* leaf agglutinin encoding gene (*ASAL*) and herbicide tolerance gene (*BAR*) were introduced into an elite cotton inbred line (NC-601) employing *Agrobacterium*-mediated genetic transformation. Cotton transformants were produced from the phosphinothricin (PPT)-resistant shoots obtained after co-cultivation of mature embryos with the *Agrobacterium* strain EHA105 harbouring recombinant binary vector pCAMBIA3300-*ASAL*-*BAR*. PCR and Southern blot analysis confirmed the presence and stable integration of *ASAL* and *BAR* genes in various transformants of cotton. Basta leaf-dip assay, northern blot, western blot and ELISA analyses disclosed variable expression of *BAR* and *ASAL* transgenes in different transformants. Transgenes, *ASAL* and *BAR*, were stably inherited and showed co-segregation in T_1_ generation in a Mendelian fashion for both PPT tolerance and insect resistance. *In planta* insect bioassays on T_2_ and T_3_ homozygous *ASAL*-transgenic lines revealed potent entomotoxic effects of ASAL on jassid and whitefly insects, as evidenced by significant decreases in the survival, development and fecundity of the insects when compared to the untransformed controls. Furthermore, the transgenic cotton lines conferred higher levels of resistance (1–2 score) with minimal plant damage against these major sucking pests when bioassays were carried out employing standard screening techniques. The developed transgenics could serve as a potential genetic resource in recombination breeding aimed at improving the pest resistance of cotton. This study represents the first report of its kind dealing with the development of transgenic cotton resistant to two major sap-sucking insects.

## Introduction

Tetraploid cotton (*Gossypium hirsutum* L.) has been the principal commercial crop and is cultivated in about 80 countries [1]. It provides livelihood for more than 180 million people with an annual contribution of $500 billion [2]. Besides being the backbone of the textile industry, cotton and its byproducts are also part of the livestock feed, seed-oil, fertilizers, paper and other consumer products [3]. Approximately, 82% of cotton productivity is jeopardized by the occurrence of various biotic stresses such as pests, weeds and pathogens [4]. Among different insect pests of cotton, bollworms, sap-sucking pests, stem, leaf and foliar feeders are the major insects which cause significant damage to the crop productivity. Sap-sucking pests, viz., jassids (*Amrasca devastans*), whiteflies (*Bemisia tabaci*) and aphids (*Aphis gossypii*), cause serious damage to the crop both directly and by acting as vectors for different pathogens [5]. Moreover, these insects are difficult to control using the conventional pesticide regimes owing to their rapid adaptation and development of resistance to the insecticides.

Crop protection plays a vital and integral role in the modern-day agricultural production. In cotton, the plant features desired for durable resistance to various pests make it difficult to obtain pest resistant genotypes. For example, increased trichome density enhances the resistance to jassids, but on other hand renders plants more susceptible to bollworms [6]. Genetic enhancement of cotton through conventional breeding for incorporation of insect resistance met with little success due to the non-availability of genotypes with the required level of resistance. Although chemical control of insect pests is an effective option, most often it is expensive and depends mainly on the weather conditions. Despite the measures for controlling insects by the application of chemical pesticides, about 30% of cotton production is lost to various pests [4]. Growing awareness of the problems associated with pesticide applications and lack of desired traits in the cotton germplasm heightened the interest to rely upon and adopt the transgenic technology [7].

Significant advances made in regeneration protocols and gene delivery methods have facilitated the introduction of beneficial genes for various traits, viz., insect resistance, improved fiber quality, herbicide and drought tolerance into cotton. Transgenic technology is known to offer unique opportunities for effective management of diverse pest populations prevailing in different agro-climatic zones [8]. Transgenic cotton, expressing genes coding for insecticidal crystalline proteins from *Bacillus thuringiensis* (*Bt*), has achieved remarkable success both economically and ecologically [9]. Although the *Bt* cotton, with reduced usage of broad spectrum insecticides, has provided effective plant protection against major lepidopteran insects, yet it shows susceptibility towards sucking pests [10] **.** Moreover, the increased pest status of numerous homopteran species and their increased resistance to chemical insecticides provided impetus for the development of alternative management strategies [11].

Plants are known to serve as sources of non-*Bt* insecticidal proteins such as lectins and protease inhibitors. Lectins are carbohydrate-binding proteins that specifically recognize glycans of glycoproteins, glycolipids or polysaccharides with high affinity, and thus mediate various biological processes, viz., cell-cell and host-pathogen interactions, besides innate immune responses [12]. The possible mechanism of the lectin toxicity in insects seems to involve the binding of lectins to the brush border membrane vesicle receptors of gut epithelial cells, thereby causing disruption of cell function and mortality [13]. Lectins derived from diverse plant species have been found to provide effective protection against several insect pests when expressed in transgenic plants [14], [15], [16], [17]. Mannose-specific *Allium sativum* leaf agglutinin genes, *ASA* and *ASAL*, when expressed in rice conveyed marked resistance against homopteran pests [17], [18]. Also, pyramided rice lines harbouring *ASAL* and *Galanthus nivalis* (*GNA*) lectin genes revealed high-level resistance against major sap-sucking pests [8].

The present investigation deals with the ectopic expression of *ASAL* gene in an elite inbred cotton line used in hybrid production through *Agrobacterium*-mediated genetic transformation. Molecular evidences suggest the stable integration of *ASAL* into the genome of cotton plants and its variable expression. Stable transgenic lines expressing ASAL showed explicit resistance against two major cotton sap-sucking insects, viz., jassid and whitefly.

## Results

### Genetic Transformation and Production of Transgenic Cotton Plants using pCAMBIA3300-*ASAL*-*BAR* Plant Expression Vector

**Figure 1:**
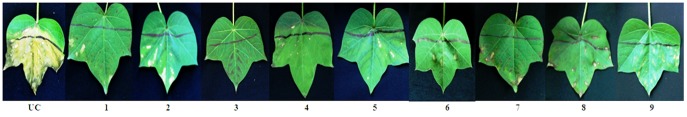
Basta treated leaves of cotton transformants showing tolerance to the herbicide. UC: Untransformed control leaf showing damage to the herbicide. 1–9: Leaves of different cotton transformants showing tolerance to the herbicide.

The *ASAL* plant expression cassette, comprising CaMV35S promoter, *ASAL* coding region and *nos* terminator, was cloned at HindIII site of pCAMBIA3300-*BAR* binary vector. The recombinant clone was designated as pCAMBIA3300-*ASAL*-*BAR*. Later, it was introduced into *Agrobacterium* strain (EHA105) and was used for the genetic transformation experiments. To insert *ASAL* and *BAR* genes into the cotton genome, embryos derived from mature seeds were co-cultivated with the *Agrobacterium* strain EHA105 harbouring pCAMBIA3300-*ASAL*-*BAR*. Selection and regeneration of cotton plants from the co-cultivated embryos are shown in Figure S1. A total of 21 putative transformants were obtained from 10305 embryos infected, after two rounds of selections on PPT medium. Out of these, 9 transformants were selected for further analyses based on their tolerance to herbicide 0.2% Basta (Figure 1).

### Molecular Analysis of Primary (T_0_) Transgenic Plants

**Figure 2:**
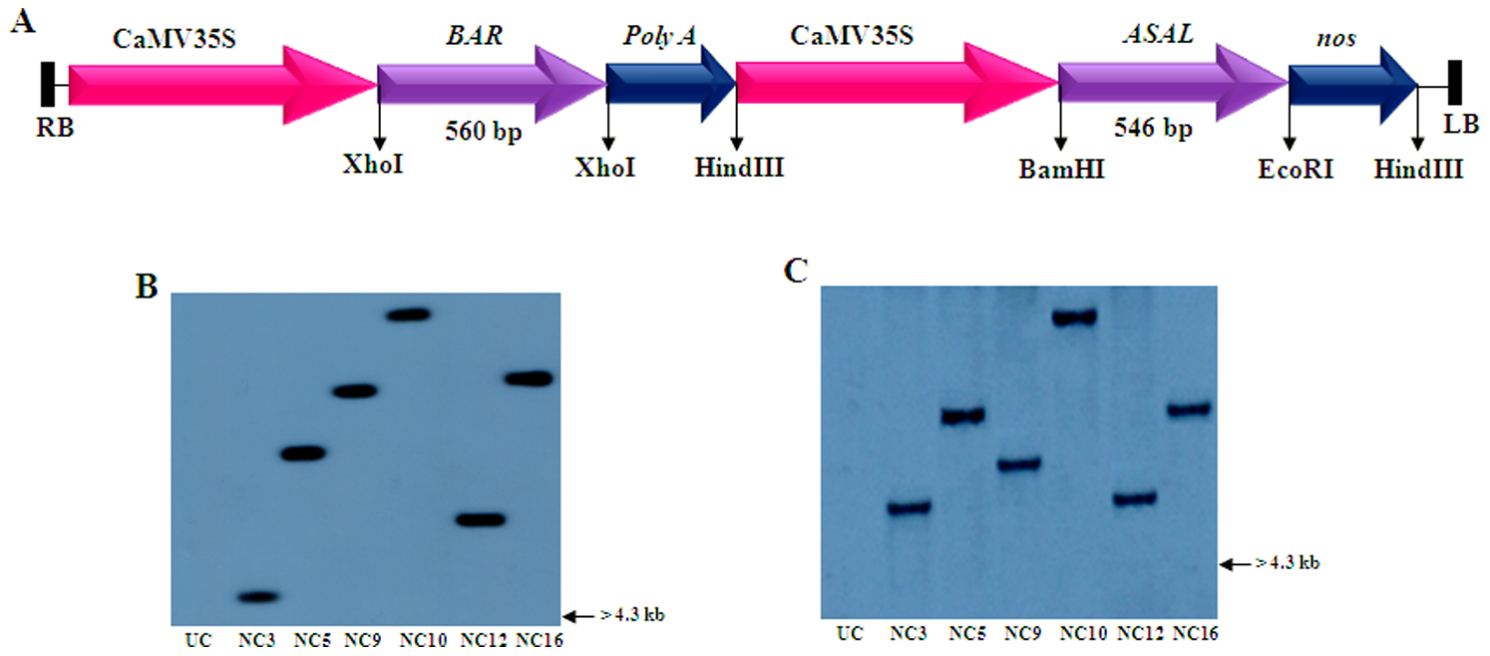
Southern blot analyses of transgenic cotton plants. (A) Restriction map of T-DNA region of pCAMBIA3300 containing *ASAL* and *BAR* expression units. (B) Genomic DNA digested with EcoRI and probed with *ASAL* coding sequence. (C) Genomic DNA digested with HindIII and probed with *BAR* coding sequence. Lane UC: DNA from untransformed control plant. Lanes NC3, NC5, NC9, NC10, NC12 and NC16: DNA from different transgenic lines.

**Figure 3:**
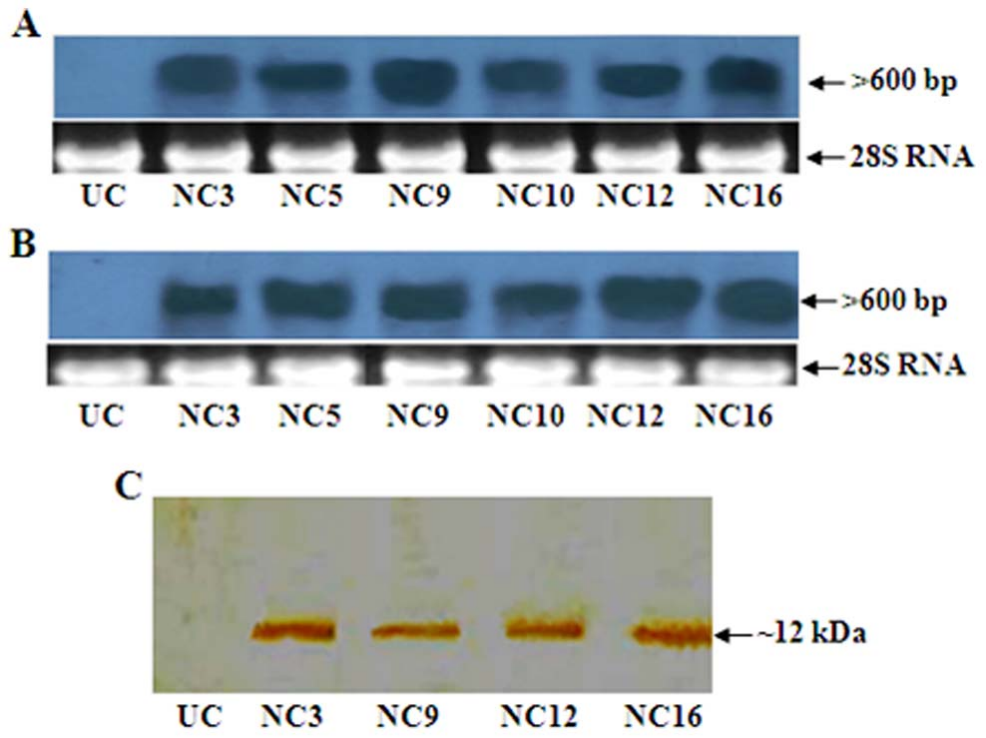
Northern and western blot analyses for the expression pattern of transgenes in transgenic cotton lines. (A) RNA probed with *ASAL* coding sequence. (B) RNA probed with *BAR* coding sequence. (C) Protein extracts from cotton plants treated with anti-ASAL antibodies. Lane UC: Samples from untransformed control plants. Lanes NC3, NC5, NC9, NC10, NC12 and NC16: Samples from different transgenic lines. Ethidium bromide stained 28S RNA band is shown under northern blots for amount of RNA loading.

Genomic DNA was isolated from the Basta tolerant transgenic cotton plants as well as from the untransformed control plants. PCR analysis of transgenic cotton plants, using gene and terminator-specific primers, showed amplification of ∼750 bp and ∼700 bp fragments, representing *ASAL-nos* and *BAR-PolyA* sequences, respectively, while control plants failed to show such amplifications (Figure S2). Southern blot analysis of genomic DNA from Basta tolerant and PCR positive plants, digested with EcoRI and HindIII separately, and probed with *ASAL* and *BAR* coding sequences, showed different hybridizable bands of >4.3 kb (Figure 2). Conversely, the untransformed control plants failed to show any hybridizable band under identical conditions. Northern blot analysis was performed using the total RNA isolated from the Southern-positive plants to assess the expression levels of *ASAL* and *BAR* genes in different transgenic cotton lines. The results revealed the presence of >600 bp hybridizable bands of varied intensity in different transgenic lines (Figure 3A and 3B). Western blot analysis of leaf extracts from transgenic cotton plants showed the presence of a polypeptide of **∼**12 kDa band corresponding to the ASAL protein when treated with anti-ASAL antibodies (Figure 3C); while, no such band was noticed in the untransformed control plants. The level of ASAL expression in transgenic plants was determined by ELISA, and the amount of ASAL among transformants ranged between 0.45% and 1.92% of the total soluble proteins.

### Inheritance Pattern of *ASAL* and *BAR* Genes in T_1_ Generation

To investigate the inheritance pattern of transgenes, selfed seed collected from the primary (T_0_) transformants of NC-1, NC-3, NC-5, NC-7, NC-8, NC-9, NC-10, NC-12 and NC-16 were germinated and tested for their tolerance to PPT as well as resistance to jassid and whitefly insects. Segregation analyses of transgenes (*BAR*/*ASAL*) in various T_1_ progenies, showed a monogenic ratio of 3 tolerant (T) and resistant (R): 1 susceptible (S) plants for PPT and insect resistance (Table S1 and Figure S3). Out of these nine transformants tested, NC-_3-1-8_, NC-_9-1-15_, NC-_12-1-11_ and NC-_16-1-6_ disclosed higher level of resistance (1 to 2 score) as well as higher amount of ASAL protein (0.7 to 1.9%). These transgenic lines were healthy and were found similar to that of untransformed control plants for various morphological characters with normal seed fertility.

### Identification of Homozygous Transgenic Cotton Lines using PPT Test in T_2_ Generation

Homozygous transgenic cotton lines were identified in T_2_ generation employing the PPT tolerance test. Selfed seed of 64 T_2_ progenies derived from NC-_3-1-8_, NC-_9-1-15_, NC-_12-1-11_ and NC-_16-1-6_ lines, when germinated on MS medium containing PPT, showed 12 T_2_ progenies with complete germination, 39 progenies segregated into 3∶1 ratio, and the seeds of 13 progenies failed to show germination. Non segregating lines for germination on PPT medium were selected as homozygous lines for further studies. Moreover, homozygosity of selected transgenic lines was validated through Basta leaf dip assay. All the plants of selected homozygous lines invariably exhibited tolerance to 0.2% Basta, and were employed for insect bioassays.

### Impact of ASAL on Jassid and Whitefly

**Figure 4:**
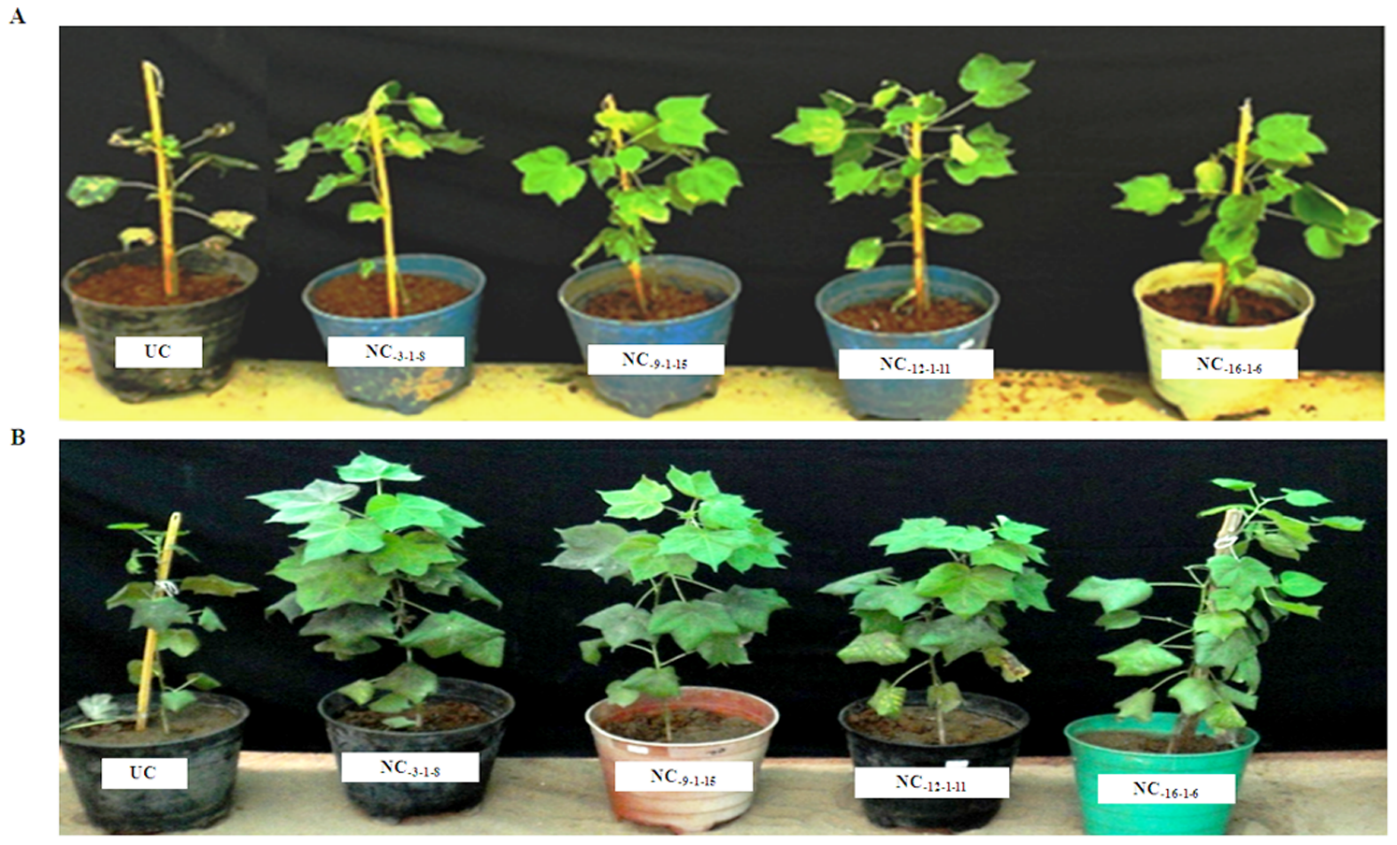
Jassid and whitefly bioassays on homozygous transgenic plants of cotton. (A) 45-day old transgenic lines along with untransformed control plant infested with jassid. (B) 45-day old transgenic lines along with untransformed control plant infested with whitefly. UC: untransformed control plants showing susceptibility against jassid and whitefly infestation with complete damage (4 on a 1 to 4 scale). NC_3-1-8_, NC_9-1-15_, NC_12-1-11_ and NC_16-1-6_: Transgenic cotton lines expressing ASAL showing significant resistance (1 to 2 on a 1 to 4 scale) against jassid and whitefly infestation with minimal plant damage.

**Figure 5:**
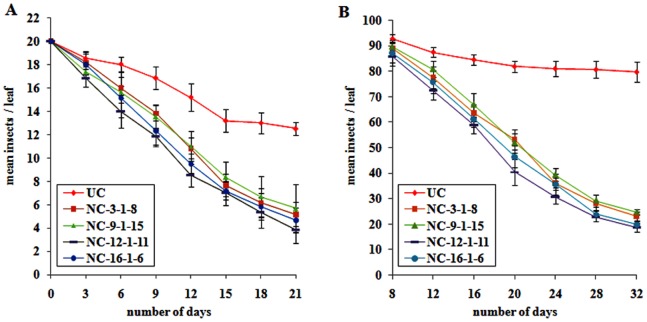
Survival of jassid and whitefly insects on transgenic cotton lines expressing ASAL. (A) Twenty jassid nymphs were released onto each single leaf enclosure and nymphal survival was recorded at every 3-day interval upto 21 days. (B) One adult whitefly pair (1male:1female) was released onto each single leaf enclosure and nymphal survival was recorded from eighth day onwards for every 4-day interval upto 32 days. Transgenic lines NC_3-1-8_, NC_9-1-15_, NC_12-1-11_ and NC_16-1-6_ are depicted by square, triangle, rectangle and circle, respectively. Untransformed control (UC) plants are depicted by diamond. Bioassays were carried out with five replications and were repeated thrice. Differences between control and transgenic plants were significant at p<0.0001. Bars indicate mean ± SE.

**Figure 6:**
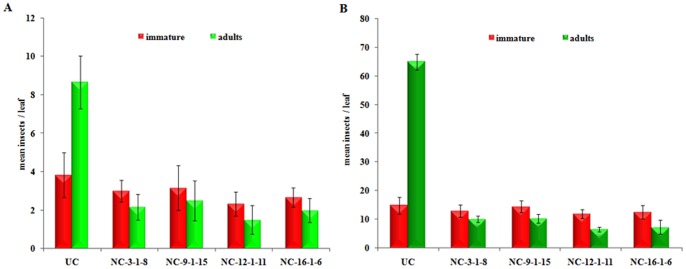
Effect of ASAL on the development of jassid and whitefly insects. (A) Twenty jassid nymphs were released onto each single leaf enclosure. After 21 days, the number of nymphs reached adult stage and the number of nymphs remained immature were plotted on the graph. (B) One adult whitefly pair was released onto each single leaf enclosure and after 32 days the number of nymphs reached adult stage and the number of nymphs remained immature were plotted on the graph. UC: untransformed control plants. NC_3-1-8_, NC_9-1-15_, NC_12-1-11_ and NC_16-1-6_: Different transgenic cotton lines expressing ASAL. Bioassays were carried out with five replications and were repeated thrice. Differences between control and transgenic plants were significant at p<0.0001. Bars indicate mean ± SE.

Comprehensive *in planta* bioassays were performed for the insecticidal activity of ASAL using T_2_ and T_3_ (homozygous) transgenic cotton lines against two major sap-sucking pests. Homozygous transgenic cotton lines of NC-_3-1-8_, NC-_9-1-15_, NC-_12-1-11_ and NC-_16-1-6_ expressing ASAL showed significant resistance with minimal plant damage and exhibited a score of 1 to 2 on a 1 to 4 scale. On the other hand, untransformed control plants showed complete damage with a score of 4 on 1 to 4 scale (Figure 4A and 4B). Further insect bioassays such as mortality, developmental delay and fecundity analysis were carried out on these selected plants. During the entire 21-day bioassay period, the survival of jassids on transgenic cotton plants was significantly reduced to 5.17±1.06, 5.67±2.06, 3.83±1.16 and 4.66±1.03 insects/leaf on NC_-3-1-8_, NC_-9-1-15_, NC_-12-1-11_ and NC_-16-1-6_ lines, respectively, as compared to 12.50±0.54 insects/leaf on untransformed control plants (Figure 5A). Likewise, the survival of whitefly on transgenic cotton plants was significantly reduced to 23.16±1.72, 24.50±1.04, 18.66±1.75 and 19.83±1.16 insects/leaf on NC_-3-1-8_, NC_-9-1-15_, NC_-12-1-11_ and NC_-16-1-6_ lines, respectively, compared to 79.66±3.88 insects/leaf on control plants by the end of 32-day bioassay period (Figure 5B). The survival of jassid and whitefly nymphs was reduced by ∼55 to 69% and ∼68 to 77%, respectively, as compared to that of untransformed control plants (Figure 5). Nymphs of jassid and whitefly fed on transgenic and untransformed control plants were monitored for the effect of ASAL on their growth and development. Among jassid survivors, ∼39 to 44% could reach the adult stage on different transgenic lines, compared to ∼69% adults noticed on the control plants (Figure 6A). From whitefly survivors, only ∼35 to 43% could reach the adult stage on transgenic lines while ∼81% adults were found on the control plants (Figure 6B).

**Figure 7:**
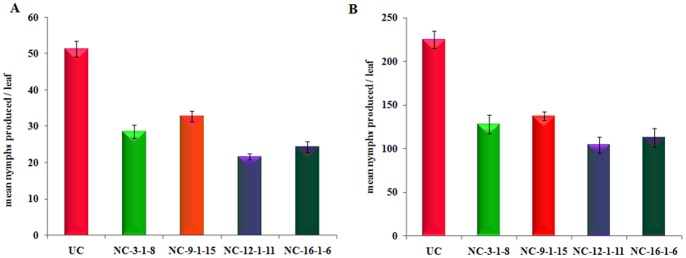
Effect of ASAL on the fecundity of jassid and whitefly insects. (A) Total number of nymphs produced by three pairs of adult jassid insects fed on untransformed control and transgenic plants were counted and plotted on the graph. (B) Total number of nymphs produced by five pairs of adult whitefly insects fed on untransformed control and transgenic plants were counted and plotted on the graph. UC: untransformed control plants. NC_3-1-8_, NC_9-1-15_, NC_12-1-11_ and NC_16-1-6_: Different Transgenic cotton lines expressing ASAL. Bioassays were carried out with five replications and were repeated thrice. Differences between control and transgenic plants were significant at p<0.0001. Bars indicate mean ± SE.

Effect of ASAL on the fecundity of jassid and whitefly was assessed by estimating the total number of nymphs produced by the insects fed on transgenic cotton plants. A mean number of 28.50±1.87, 32.83±1.47, 21.66±0.81 and 24.33±1.50 jassid nymphs/leaf were recorded on NC_-3-1-8_, NC_-9-1-15_, NC_-12-1-11_ and NC_-16-1-6_ plants compared to 51.33±2.16 nymphs/leaf on the untransformed control plants (Figure 7A). Compared to untransformed controls, the nymphal production was reduced to ∼36 to 57% on different ASAL cotton transformants. Similarly, for whitefly a mean number of 128.0±10.67, 137.33±5.04, 104.16±9.32 and 112.5±10.70 nymphs/leaf were observed on NC_-3-1-8_, NC_-9-1-15_, NC_-12-1-11_ and NC_-16-1-6_ plants in comparison with 224.66±9.97 nymphs/leaf on the control plants (Figure 7B). Compared to the untransformed controls, the nymphal production on different ASAL transformants was reduced to ∼38 to 53%.

## Discussion

Abnormal climate changes are known to reduce the productivity of diverse crop plants worldwide. To overcome this challenge, it is essential to have a range of options and tools for sustainable crop production. Genetic engineering of crops could contribute to the production of resistant varieties since it provides scope to introduce different candidate genes into elite crop varieties [19]. Homopteran insects cause severe damage to various crop plants and are difficult to control by applying insecticides due to their unique feeding habits and fast multiplication. Moreover, sap-sucking pests are not controlled by *Bt* toxins. Plants have different protective mechanisms against insects and the most important components are proteins such as protease inhibitors and lectins [17]. The extensively studied anti-insect plant lectins are the mannose-binding lectins, viz., GNA and ASAL from *Galanthus nivalis* and *A*. *sativum*, respectively. In our ongoing efforts to validate the entomotoxic effects of GNA and ASAL against different sap-sucking pests, genes encoding these proteins were overexpressed in rice and cotton. Transgenic rice lines engineered with these genes conferred substantial resistance against major sap-sucking pests [8], [14], [17], [20], [21]. The present study mainly deals with the constitutive expression of *ASAL* gene in an elite cotton inbred line and its evaluation against two major sucking pests. *In planta* bioassays, employing standard screening techniques that reflect situations prevailing in the field, revealed strong entomotoxic effects of transgenically expressed ASAL against jassid and whitefly of cotton.

Employing the protocols optimized in our laboratory, *ASAL* and *BAR* expression cassettes were introduced into the cotton inbred line NC-601 using *Agrobacterium* binary vector pCAMBIA3300-*ASAL*-*BAR* (Figure 2A). Molecular analyses of Basta tolerant plants confirmed the stable integration of *ASAL* and *BAR* genes into the cotton genome. Presence of >4.3 kb hybridizable bands with *ASAL* probe, in EcoRI digested DNA of transformants, suggests the independent nature of transgene integration in the primary transformants of cotton (Figure 2). It has been established that multiple copies of transgene(s) often result in the co-suppression and gene silencing [22], [23]. Single copy integration of transgene(s) is essential to achieve predictable patterns of inheritance and to eliminate the problem of gene silencing in the transgenic plants [24]. Northern blot analysis clearly showed the variable expression of *ASAL* and *BAR* genes in the primary transgenic plants as evidenced by varied intensity of the hybridizable band of >600 bp (Figure 3). Furthermore, western blot analysis of transgenic plants confirmed the stable expression of the *ASAL* gene at the protein level (Figure 3C). Marked variation observed in the amount of ASAL (0.45% to 1.92%), in different transformants by ELISA analysis, amply suggests that the transgene is integrated randomly at different transcriptionally active sites in the cotton genome.

To establish the definitive transgenic nature of primary transformants, the inheritance pattern of transgenes was analyzed in the T_1_ and T_2_ generations employing PPT test. In T_1_ generation, transgene segregation conformed to the monogenic ratio (3 tolerant: 1 susceptible) for PPT tolerance, testifying that these genes are stably integrated into the cotton genome (Table S1). Similarly, *in planta* insect bioassays in T_1_ generation revealed a 3 resistant: 1 susceptible ratio against jassid and whitefly insects with a score of 1 to 2 on a 1 to 4 scale. The PPT test and insect bioassays disclosed the co-segregation of *BAR* and *ASAL* transgenes (Table S1 and Figure S3). Four homozygous transgenic cotton lines, viz., NC_-3-1-8_, NC_-9-1-15_, NC_-12-1-11_ and NC_-16-1-6_ were selected in T_2_ generation employing PPT test and were used for further studies. Comprehensive *in planta* bioassays performed on selected homozygous *ASAL*-cotton lines divulged decreased insect survival, development and fecundity, thereby minimizing the damage caused by these pests.

Jassid bioassays on single leaf confinement studies disclosed 19.15 to 28.35% of nymphal survival on *ASAL*-cotton transformants as compared to 62.50% on untransformed controls. Compared to untransformed controls, nymphal survival was reduced from ∼55 to 69% in different *ASAL*-cotton transformants (Figure 5A). Among the survived nymphs ∼69% reached adult stage after 9 to 12 days on untransformed control plants, whereas only ∼39 to 44% nymphs developed into adults after 18 to 21 days on transgenic plants (Figure 6A). The delay in reaching adulthood of insects fed on transgenic plants could be due to delayed moulting which contributed to prolonged life cycle. Furthermore, fecundity assays on *ASAL*-cotton lines showed marked decline in the nymphal production by ∼36 to 57% on transgenic plants compared to the untransformed controls, owing to marked decreases in the fecundity of the insects (Figure 7A). The overall results amply indicate that the ASAL expressing transgenic cotton exhibit a distinctly higher-level of resistance against jassids when compared to that of untransformed control plants.

*In planta* whitefly bioassays on single leaf confinement revealed the nymphal survival of 21.78% to 29.69% on different *ASAL*-cotton transformants as compared to 85.81% on untransformed controls (Figure 5B). Compared to untransformed controls, nymphal survival was reduced from ∼68 to 77% on different *ASAL*-cotton transformants. Among the survived nymphs, ∼81% reached adults in ∼12 days on untransformed control plants while only ∼35 to 43% nymphs developed into adults over a period of 28 to 32 days on transgenic plants (Figure 6B), owing to the high entomotoxic effect of ASAL which is responsible for delayed moulting and prolonged life cycle. Furthermore, fecundity assays on *ASAL*-cotton lines revealed substantial decline in the whitefly nymphal production by ∼38 to 53% on transgenic lines as compared to untransformed controls, indicating marked decreases in the fecundity (Figure 7B). The overall insect bioassay results suggest the high antifeedant and entomotoxic effects of ASAL against jassids and whitefly insects.

Plant lectins are known to inhibit the nutrient absorption and cause severe disruption of the epithelial cells in the midgut of insects by stimulating endocytosis resulting in disorganization and elongation of the striated brush border microvilli, leading to the swelling of epithelial cells and complete closure of the gut lumen [25]. Earlier, it was reported that over-expression of mannose-specific lectins (ASAL and GNA) in transgenic rice lines proved more effective in reducing survival, fecundity and feeding ability of major sap-sucking insects, viz., BPH, GLH and WBPH [8], [14], [17], [18], [21]. Furthermore, transgenic rice plants expressing GNA and ASAL showed variable levels of entomotoxic effects on these sucking pests [8].

Garlic lectins bind to several proteins such as cadherin-like proteins, polycalins, sucrase, cytochrome P450 and symbionin in the midgut of insects through glycan mediated interactions [26]-[28], which affects normal metabolism and cell functions leading to rapid feedback response on the feeding behaviour of insect. It was also reported that transgenic crops expressing garlic lectins (ASA and ASAL) could confer resistance against various lepidopteran and homopteran insects, viz., *Nilaparvata lugens*, *Nephotettix virescens*, *Sogatella furcifera, Spodoptera littoralis*, *Lipaphis erysimi* and *Aphis craccivora* [8], [17], [29]-[31]. Further, it was reported that the expression of *Dioscorea batatas* tuber lectin in rice and tobacco reduced the survival of BPH and aphid by 30% and 60%, respectively [32], [33]. Garlic lectins serve as promising candidate molecules for the development of transgenic crops against sucking pests as they are from edible source, and as such may not pose any biosafety problem.

*In planta* insect bioassays, reported herein, were carried out on *ASAL* transgenic cotton lines adopting standard screening techniques followed in the conventional cotton breeding for selection of insect resistant plants. Transgenic plants expressing ASAL protein revealed potent entomotoxic effects of garlic lectin on two major sucking pests of cotton, viz., jassids and whitefly as evidenced by reduction in the survival, development and fecundity of the insects. The developed cotton transgenics appear promising for commercial cultivation and also serve as a potential genetic resource in recombination breeding aimed at developing resistance to sucking pests. Moreover, these lines can be crossed with *Bt* transgenics for production of cotton resistant to both sucking and chewing pests.

## Materials and Methods

### Construction of Binary Vector Containing *ASAL* and *BAR* Expression Cassettes

The 1.6 kb *ASAL* plant expression cassette (CaMV35S-*ASAL-nos*) was excised with HindIII digestion from pSB11 *BAR* intermediate vector [17], and cloned into HindIII site of pCAMBIA3300 binary vector of *Agrobacterium* containing *BAR* gene as a plant selectable marker (CaMV35S-*BAR*-*PolyA*). The recombinant binary vector, containing *BAR* and *ASAL* expression cassettes, was maintained in HB101 cells and mobilized into *A*. *tumefaciens* strain EHA105 by tri-parental mating [34] using the helper vector pRK2013.

### *Agrobacterium*-mediated Transformation and Regeneration of Transgenic Plants

Genetic transformation experiments were carried out using the inbred line of cotton NC-601 and *Agrobacterium* strain EHA105 harbouring pCAMBIA3300-*ASAL*-*BAR* binary vector. Seeds of NC-601 obtained from M/s. Nuziveedu Seeds Pvt. Ltd, Hyderabad (India) were delinted with H_2_SO_4_ and thoroughly rinsed under running tap water for ∼20 min and kept for drying. The dried seeds were surface-sterilized with 0.1% HgCl_2_ for 10–12 min, followed by three washes with sterile distilled water for 10 min each, and soaked in sterile water at 30±2°C for 4 h. Later, embryos were aseptically separated from the imbibed seeds and were used for genetic transformation. *Agrobacterium* culture was initiated by inoculating a single colony into 6 ml YEP medium containing 50 mg/l kanamycin and 10 mg/l rifampicin, and allowed to grow for 24 h at 29°C with 225 rpm. The bacterial culture was pelleted at 3500 rpm and resuspended in 12 ml of PIMII medium [35] and incubated for 16 h at 29°C. For co-cultivation, 150-200 embryos were transferred into 50 ml PIMII medium in a conical flask and 5 ml of *Agrobacterium* culture growing in PIMII medium was added, and left on the shaker at 225 rpm for 72 h at 29°C in dark. The infected embryos were later washed thrice in Murashige and Skoog (MS) [36] basal medium with 250 mg/l cefotaxime before transferring to the first selection medium (MS basal +0.125 mg/l BAP +0.05 mg/l NAA +5 mg/l PPT). The shoots obtained from the surviving embryos on the selection medium were excised and were transferred to second selection medium (MS basal +0.05 mg/l kinetin +6 mg/l PPT) for four weeks. The survived shoots were transferred to the rooting medium (MS basal +1.5 mg/l IBA) and the rooted plantlets were transferred to the pots, and allowed to grow to maturity in the glasshouse. Forty five day old untransformed controls and putative transformants were tested for their tolerance to the herbicide Basta (Bayer CropScience Ltd.). The herbicide test was conducted by dipping the half of the leaf into 0.2% Basta solution. The leaves treated with herbicide were monitored after 72 h to see the damage caused by Basta.

### PCR and Southern Blot Analyses

The genomic DNA was isolated from the Basta tolerant and untransformed control plants as described by Paterson et al [37]. PCR analysis was carried out using the primers corresponding to expression cassettes of *ASAL* F, 5′-ATGGGTCCTACTACTTCATCTCCT-3′ and *nos* R, 5′-GTTTGCGCGCTATATTTGTT-3′, and *BAR* F, 5′-CTACCATG AGCCCAG AACG-3′ and *PolyA* R, 5′-AGCGAAACCCTATAAGAACCC-3′. DNA from the untransformed control plants was used as negative control and the binary vector was used as a positive control. For Southern blot analyses about 20 µg of genomic DNA was used. Genomic DNA digested independently with EcoRI and HindIII, probed with *ASAL* and *BAR* coding regions, were used to assess the independent nature of transformants. Digested genomic DNAs were separated by electrophoresis on a 0.8% agarose gel, and subsequently transferred to the N^+^ Nylon membrane and then fixed by exposing to UV (1200 µJ for 60 s) in an UV cross linker [38]. The blots were hybridized with the 560 bp *BAR* and 546 bp *ASAL* coding regions as probes which were prepared by the AlkPhos Direct Labeling System (Cat. No. RPN3680, GE Healthcare) following the supplier’s instructions. Membrane washing and detection procedures were performed according to the supplier’s instructions.

### Northern Blot Analysis

Total RNA isolated from the leaves of Southern positive and untransformed control plants was used for northern blot analysis as described by Wan and Wilkins [39]. About 12 µg of total RNA was separated on 1.4% denaturing agarose gel and was blotted onto nylon membrane and fixed by exposing to UV (1200 µJ for 60 s) in an UV cross linker. The 546 bp *ASAL* and 560 bp *BAR* coding regions were used as probes for northern hybridization. Hybridization, membrane washing and detection procedures were performed following the supplier’s instructions (AlkPhos Direct Labeling System; GE Healthcare).

### Western Blot Analysis of Transgenic Cotton Plants

Samples of transgenic and untransformed control leaf tissue were homogenized in buffer consisting of 100 mM potassium phosphate buffer (pH 7.8), 1 mM EDTA (pH 8.0), 1% Triton X-100, 10% glycerol and 1 mM DTT. The extract was centrifuged at 12,000 rpm for 20 min at 4°C, and the supernatant was collected. Protein samples (10 µg) were subjected to 15% SDS-PAGE according to Laemmli [40]. Following electrophoresis, the separated proteins were transferred onto Hybond-P membrane (Amersham) by electroblotting [41]. After protein transfer, the membrane was blocked by incubating in PBS solution containing 10% non fat dried milk and 0.1% Tween 20 for 2 h at room temperature. The membrane was probed with polyclonal rabbit anti-ASAL serum (1∶3000 dilution) and goat anti-rabbit IgG horse-radish peroxidase conjugate (GENEi) as secondary antibody (1∶3000 dilution). The membrane was washed and analyzed with saturated benzidine solution containing 20% ammonium chloride and 0.1% H_2_O_2_.

### ELISA Analysis

Wells of the microtitre plate were coated with 1 µg of crude protein extract of untransformed control as well as transgenic plants and kept for overnight at 37°C. The wells were washed thrice with 20 mM PBS containing 0.05% Tween 20 and were blocked with 10% non-fat dried milk for 2 h at 37°C, subsequently washed six times with PBS-T. The primary antibody (1∶3000) was added to the wells and incubated for 2 h at 4°C. After incubation, the wells were washed thrice with PBS and incubated with secondary antibody (1∶3000) for 1 h at room temperature. The plates were washed thrice with PBS and 0.001% 3, 3′, 5, 5′- tetramethylbenzidine (TMB) substrate in 0.05M phosphate citrate buffer was added along with 0.1% H_2_O_2_ and kept in dark for 10 min. The reaction was stopped by 1N H_2_SO_4_ and the absorbance was recorded on ELISA reader at 450 nm.

### Phosphinothricin (PPT) Tolerance Test

PPT test was performed to study the inheritance pattern of transgenes as well as identification of homozygous transgenic cotton lines. Selfed seed collected from T_1_, T_2_ and T_3_ transgenic lines were delinted and surface-sterilized with 0.1% HgCl_2_ as mentioned earlier. Seeds were allowed to germinate on the MS medium containing 6 mg/l PPT and placed under light. Data were recorded 7 days after seed germination.

### Insect Rearing

Insect populations of jassids and whiteflies were maintained on cotton and bendhi, respectively, under controlled conditions (Temperature 29–31°C and humidity 70–75%) in the poly-house. Pre-mated females of jassid and whitefly oviposited separately and the nymphs after attaining the desired age were used for different experiments.

### Insect Bioassays

*In planta* insect bioassays were carried out under standard controlled conditions in the poly-house employing standard screening techniques followed in conventional breeding for selection of insect resistant plants. T_1,_ T_2_ and T_3_ (homozygous) *ASAL*-cotton transformants along with untransformed controls were grown and enclosed in an insect proof mylar cage for insect bioassays. Forty five-day old plants were challenged with jassid/whitefly and after three weeks, when the untransformed controls exhibited complete damage, the experiments were terminated. The resistance exhibited by the transgenic plants against jassids and whitefly was evaluated based on a scale of 1 to 4 [42], [43].

### Survival and Development Assays

Forty five-day old homozygous transgenic cotton lines and untransformed control plants were used to assess insect mortality/survival following the no choice method. For this study, 20 jassid nymphs were released onto each single leaf enclosure made using muslin cloth. Survival of jassid nymphs was monitored and observations were recorded after every 3-day interval upto 21days. In case of whitefly, one adult pair (1male:1female) was released onto each single leaf and allowed for eight days. Later, the nymphal survival was recorded after every 4-day interval upto 32 days. Delay in the development of insects was also observed by scoring the number of adults and insects still in nymphal stage.

### Fecundity Assays

Forty five-day old homozygous transgenic cotton lines and untransformed control plants were tested to assess the efficacy of ASAL on the fecundity of jassid and whitefly populations. For this study, 3 pairs of jassids/5 pairs of whitefly adults in a 1male:1female ratio were pooled and released into a confined leaf to avoid differences in the nymphal production based on the sex ratio. The total number of nymphs emerged were counted after 21 and 32 days for jassids and whiteflies, respectively. All the bioassay experiments were carried out with five replications and were repeated thrice. Data analysis was done using MS-Excel 2007 software. Differences between the mean values were subjected to unpaired t*-*test.

## Supporting Information

Figure S1 **Genetic transformation and production of transgenic cotton plants.**
(DOCX)

Figure S2 **PCR analyses of putative cotton transformants.**
(DOCX)

Figure S3 **Inheritance pattern of** ***BAR*** **gene in T_1_ generation.**
(DOCX)

Table S1 **Inheritance pattern of** ***ASAL*** **-** ***BAR*** **transgenes, and jassid and whitefly bioassays in T_1_ generation.**
(DOCX)
