# Supplementary material for: Development of Transgenic Cotton Lines Expressing Allium sativum Agglutinin (ASAL) for Enhanced Resistance against Major Sap-Sucking Pests
Source: PLoS One. 2013 Sep 4;8(9):e72542. doi: 10.1371/journal.pone.0072542 (PMC3762794; doi:10.1371/journal.pone.0072542)
Supplement: Figure S1 — Genetic transformation and production of transgenic cotton plants. (DOCX) [file pone.0072542.s001.docx]

(B)

(F)

(E)

(A)

(D)

(C)


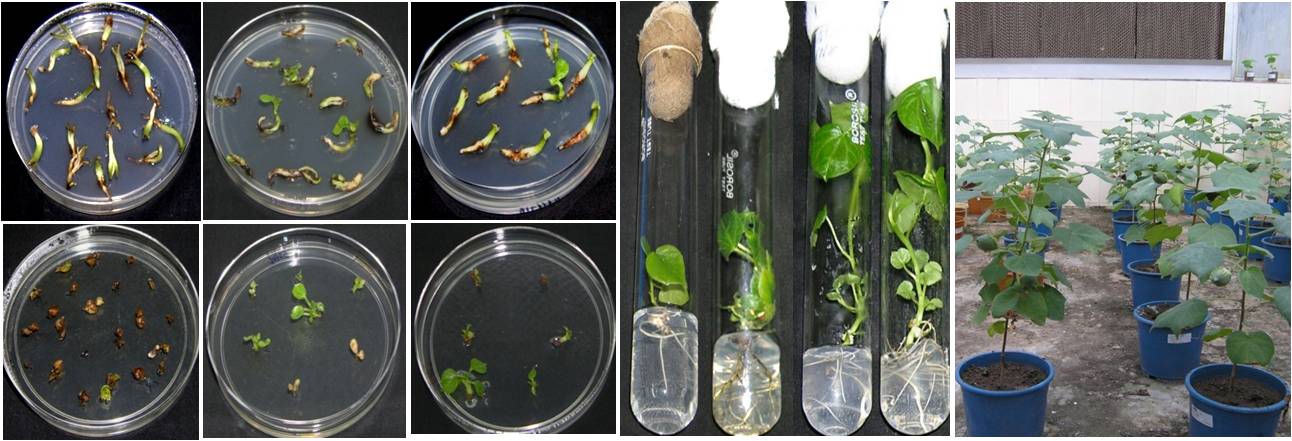


**Figure S1. Genetic transformation and production of transgenic cotton plants.** (A) Control embryos on selection medium containing PPT (5 mg/l). (B) Co-cultivated embryos producing shoots on the first selection medium containing PPT (5 mg/l). (C) Control shoots on selection medium containing PPT (6 mg/l). (D) Putatively transformed shoots surviving on the second selection medium containing PPT (6 mg/l). (E) Root induction from shoots on the MS medium supplemented with IBA (1.5 mg/l). (F) Putative cotton transformants growing in the glasshouse.
