# Supplementary material for: Development of Transgenic Cotton Lines Expressing Allium sativum Agglutinin (ASAL) for Enhanced Resistance against Major Sap-Sucking Pests
Source: PLoS One. 2013 Sep 4;8(9):e72542. doi: 10.1371/journal.pone.0072542 (PMC3762794; doi:10.1371/journal.pone.0072542)
Supplement: Figure S2 — PCR analyses of putative cotton transformants. (DOCX) [file pone.0072542.s002.docx]

(A)

~750 bp

1 2 3 4 5 6 7 8 9 10 11 12


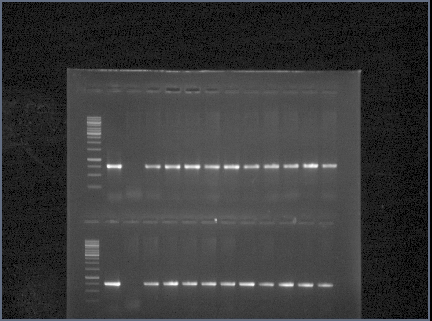


(B)

~700 bp

1 2 3 4 5 6 7 8 9 10 11 12


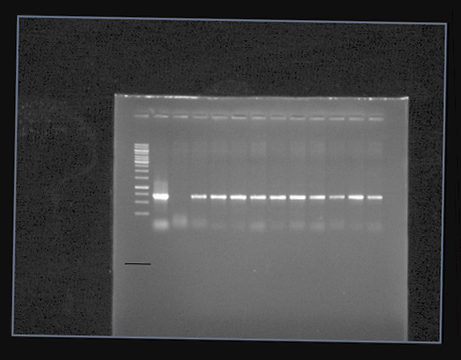


**Figure S2. PCR analyses of putative cotton transformants.** (A) Lane1: 1 kb marker. Lane 2: pCAMBIA3300-*ASAL*-*BAR* plasmid showing ~750 bp band corresponding to *ASAL* and *nos* sequence. Lane 3: Genomic DNA from untransformed control plant. Lanes: 4-12 genomic DNA from different transformants displaying amplification of *ASAL* and *nos* sequence. (B) Lane1: 1 kb marker. Lane 2: pCAMBIA3300-*ASAL*-*BAR* plasmid showing ~700 bp band corresponding to *BAR* and *PolyA* sequence. Lane 3: Genomic DNA from untransformed control plant. Lanes: 4-12 genomic DNA from different transformants displaying amplification of *BAR* and *PolyA* sequence.
