# Supplementary material for: Development of Transgenic Cotton Lines Expressing Allium sativum Agglutinin (ASAL) for Enhanced Resistance against Major Sap-Sucking Pests
Source: PLoS One. 2013 Sep 4;8(9):e72542. doi: 10.1371/journal.pone.0072542 (PMC3762794; doi:10.1371/journal.pone.0072542)
Supplement: Figure S3 — Inheritance pattern of BAR gene in T1 generation. (DOCX) [file pone.0072542.s003.docx]

(B)

(A)

(F)

(G)

(H)

(E)

(D)

(C)


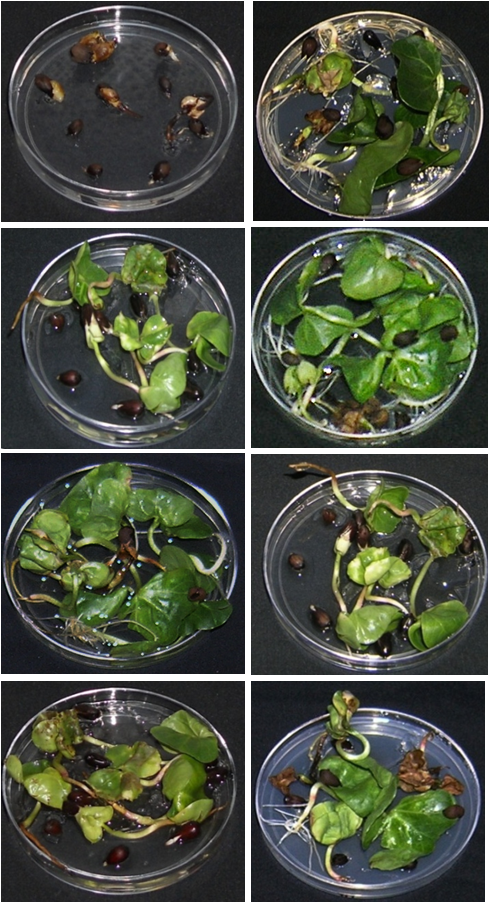


**Figure S3. Inheritance pattern of *BAR* gene in T_1_ generation.** (A) Seeds of untransformed control plant kept for germination on MS medium containing 6 mg/l phosphinothricin. (B-H) Seeds of transformants kept for germination on MS medium containing 6 mg/l phosphinothricin.
