# Supplementary material for: Development of Transgenic Cotton Lines Expressing Allium sativum Agglutinin (ASAL) for Enhanced Resistance against Major Sap-Sucking Pests
Source: PLoS One. 2013 Sep 4;8(9):e72542. doi: 10.1371/journal.pone.0072542 (PMC3762794; doi:10.1371/journal.pone.0072542)
Supplement: Table S1 — Inheritance pattern of ASAL - BAR transgenes, and jassid and whitefly bioassays in T1 generation. (DOCX) [file pone.0072542.s004.docx]

**Table S1. Inheritance pattern of *ASAL*-*BAR* transgenes, and jassid and whitefly bioassays in T_1_ generation**

| **T_1_ Progenies** | **Basta test /Bioassay** | **Total No. of plants tested** | **No. of resistant plants** | **No. of susceptible plants** | **Expected ratio** | χ**^2^-value** |
| --- | --- | --- | --- | --- | --- | --- |
| Control | Basta | 21 | 0 | 21 | -- | -- |
| NC_-1-1_ | Basta | 18 | 11 | 7 | 3:1 | 1.851 |
| NC_-3-1_ | Basta | 17 | 12 | 5 | 3:1 | 0.176 |
| NC_-5-1_ | Basta | 16 | 11 | 5 | 3:1 | 0.333 |
| NC_-7-1_ | Basta | 19 | 13 | 6 | 3:1 | 0.438 |
| NC_-8-1_ | Basta | 23 | 15 | 8 | 3:1 | 1.173 |
| NC_-9-1_ | Basta | 21 | 13 | 8 | 3:1 | 1.920 |
| NC_-10-1_ | Basta | 19 | 13 | 6 | 3:1 | 0.438 |
| NC_-12-1_ | Basta | 19 | 15 | 4 | 3:1 | 0.157 |
| NC_-16-1_ | Basta | 21 | 17 | 4 | 3:1 | 0.396 |
| Control | jassid | 19 | 0 | 19 | -- | -- |
| NC_-1-1_ | jassid | 21 | 14 | 7 | 3:1 | 0.777 |
| NC_-3-1_ | jassid | 20 | 13 | 7 | 3:1 | 1.066 |
| NC_-5-1_ | jassid | 21 | 13 | 8 | 3:1 | 1.920 |
| NC_-7-1_ | jassid | 17 | 11 | 6 | 3:1 | 0.960 |
| NC_-8-1_ | jassid | 22 | 14 | 8 | 3:1 | 1.515 |
| NC_-9-1_ | jassid | 20 | 14 | 6 | 3:1 | 0.266 |
| NC_-10-1_ | jassid | 21 | 17 | 4 | 3:1 | 0.396 |
| NC_-12-1_ | jassid | 20 | 17 | 3 | 3:1 | 1.066 |
| NC_-16-1_ | jassid | 23 | 16 | 7 | 3:1 | 0.362 |
| Control | whitefly | 20 | 0 | 20 | -- | -- |
| NC_-1-1_ | whitefly | 20 | 14 | 6 | 3:1 | 0.266 |
| NC_-3-1_ | whitefly | 19 | 12 | 7 | 3:1 | 1.421 |
| NC_-5-1_ | whitefly | 21 | 13 | 8 | 3:1 | 1.920 |
| NC_-7-1_ | whitefly | 20 | 14 | 6 | 3:1 | 0.266 |
| NC_-8-1_ | whitefly | 22 | 14 | 8 | 3:1 | 1.515 |
| NC_-9-1_ | whitefly | 23 | 19 | 4 | 3:1 | 0.710 |
| NC_-10-1_ | whitefly | 19 | 12 | 7 | 3:1 | 0.142 |
| NC_-12-1_ | whitefly | 23 | 16 | 7 | 3:1 | 0.362 |
| NC_-16-1_ | whitefly | 24 | 19 | 5 | 3:1 | 0.222 |

The inheritance pattern of transgenes showed 3:1genotypic ratio and the calculated χ2 values were significant at p value < 0.05.
